# Inflammation and cancer

**DOI:** 10.1186/s12199-018-0740-1

**Published:** 2018-10-20

**Authors:** Mariko Murata

**Affiliations:** Department of Environmental and Molecular Medicine, Mie University Graduate School of Medicine, 2-174 Edobashi, Tsu, Mie 514-8507 Japan

**Keywords:** Inflammation, Cancer, Reactive oxygen/nitrogen species, DNA damage, DNA methylation, MicroRNA, Liquid biopsy

## Abstract

Infection and inflammation account for approximately 25% of cancer-causing factors. Inflammation-related cancers are characterized by mutagenic DNA lesions, such as 8-oxo-7,8-dihydro-2′-deoxyguanosine (8-oxodG) and 8-nitroguanine. Our previous studies demonstrated the formation of 8-oxodG and 8-nitroguanine in the tissues of cancer and precancerous lesions due to infection (e.g., *Opisthorchis viverrini*-related cholangiocarcinoma, *Schistosoma haematobium*-associated bladder cancer, *Helicobacter pylori*-infected gastric cancer, human papillomavirus-related cervical cancer, Epstein-Barr virus-infected nasopharyngeal carcinoma) and pro-inflammatory factors (e.g., asbestos, nanomaterials, and inflammatory diseases such as Barrett’s esophagus and oral leukoplakia). Interestingly, several of our studies suggested that inflammation-associated DNA damage in cancer stem-like cells leads to cancer development with aggressive clinical features. Reactive oxygen/nitrogen species from inflammation damage not only DNA but also other biomacromolecules, such as proteins and lipids, resulting in their dysfunction. We identified oxidatively damaged proteins in cancer tissues by 2D Oxyblot followed by MALDI-TOF/TOF. As an example, oxidatively damaged transferrin released iron ion, which may mediate Fenton reactions and generate additional reactive oxygen species. Dysfunction of anti-oxidative proteins due to this damage might increase oxidative stress. Such damage in biomacromolecules may form a vicious cycle of oxidative stress, leading to cancer development. Epigenetic alterations such as DNA methylation and microRNA dysregulation play vital roles in carcinogenesis, especially in inflammation-related cancers. We examined epigenetic alterations, DNA methylation and microRNA dysregulation, in Epstein-Barr virus-related nasopharyngeal carcinoma in the endemic area of Southern China and found several differentially methylated tumor suppressor gene candidates by using a next-generation sequencer. Among these candidates, we revealed higher methylation rates of RAS-like estrogen-regulated growth inhibitor (RERG) in biopsy specimens of nasopharyngeal carcinoma more conveniently by using restriction enzyme-based real-time PCR. This result may help to improve cancer screening strategies. We profiled microRNAs of nasopharyngeal carcinoma tissues using microarrays. Quantitative RT-PCR analysis confirmed the concordant downregulation of miR-497 in cancer tissues and plasma, suggesting that plasma miR-497 could be used as a diagnostic biomarker for nasopharyngeal carcinoma. Chronic inflammation promotes genetic and epigenetic aberrations, with various pathogeneses. These changes may be useful biomarkers in liquid biopsy for early detection and prevention of cancer.

## Background

It is estimated that infectious diseases and chronic inflammation account for approximately 25% of cancer-causing factors [1]. Under chronic inflammation, reactive oxygen/nitrogen species (ROS/RNS) are produced from not only inflammatory cells but also epithelial cells [2]. ROS/RNS cause DNA damage in organs under inflammation, leading to cancer development. We demonstrated the importance of mutagenic DNA lesions, especially 8-oxo-7,8-dihydro-2′-deoxyguanosine (8-oxodG, also known as 8-hydroxy dG; 8-OHdG) and 8-nitroguanine, as a common cancer-causing molecular mechanism [3].

The Khon Kaen region, in the northeastern part of Thailand, is a local endemic area for liver fluke (*Opisthorchis viverrini*; OV) infection and its related cholangiocarcinoma. OV infection is considered “carcinogenic to humans” (group 1) by the International Agency for Research on Cancer (IARC). In a collaboration with Khon Kaen University, Thailand, we firstly reported 8-nitroguanine and 8-oxodG formation in the livers of hamsters treated orally with the liver fluke in an animal model for OV-related cholangiocarcinoma [4]. In our previous studies based on DNA lesions, we investigated the molecular mechanisms of cancers due to infection (e.g., OV-related cholangiocarcinoma [2, 4–6], *Schistosoma haematobium*-associated bladder cancer [7, 8], *Helicobacter pylori*-infected gastric cancer [9], human papillomavirus-related cervical cancer [10], Epstein-Barr (EB) virus-infected nasopharyngeal carcinoma [11]), and pro-inflammatory factors (e.g., asbestos [12, 13], nanomaterials [14–17], and inflammatory diseases such as Barrett’s esophagus [18, 19], and oral leukoplakia [20]). In addition, inflammation plays a role in the epigenetic alterations in cancer. In this paper, we review the roles of RNS-/ROS-mediated DNA damage and epigenetic changes in inflammation-related carcinogenesis.

## Oxidative and nitrative damage of biomacromolecules in inflammation-related carcinogenesis

### DNA damage

In our early studies on inflammation-related carcinogenesis, we detected the formation of 8-nitroguanine and 8-oxodG in inflammatory and cancer tissues by immunohistochemistry (IHC) and an electrochemical detector coupled to HPLC (HPLC-ECD). HPLC-ECD analyses also demonstrated that urinary 8-oxodG levels were significantly higher in OV-related cholangiocarcinoma patients than in OV-infested patients and healthy subjects and higher in OV-infested patients than in healthy subjects [21]. The 8-oxodG levels of OV-infested patients significantly decreased 2 months after anti-parasite therapy and were comparable with the levels in healthy subjects 1 year after treatment. Therefore, urinary 8-oxodG can be used as a convenient biomarker of inflammation-related cancer.

Various death signals including ROS and DNA damage induce apoptosis via the mitochondrial pathway. Melatonin is a candidate chemopreventive agent for inhibiting cholangiocarcinoma development. The mechanisms of anti-cancer activity by melatonin remain unclear. We examined the induction of apoptosis in human cholangiocarcinoma cell lines treated with melatonin [22]. Flowcytometric analyses revealed that melatonin increased intracellular ROS levels and induced apoptosis. Immunocytochemistry showed melatonin-induced mitochondrial DNA damage by double staining of 8-oxodG and mitoTracker (a mitochondria-specific fluorescent staining dye). Cancer cells are in a state of high oxidative stress due to their elevated metabolism, and the additional ROS from melatonin may break the balance of anti- and pro-oxidants and damage mitochondrial DNA, leading to apoptosis. The findings offer useful information for chemoprevention and therapeutic strategies.

Chronic inflammation causes various types of damage to nucleic acids, proteins, and lipids via ROS/RNS generation, resulting in tissue damage. The tissue injury may activate progenitor/stem cells for tissue regeneration. Stem cells are damaged by ROS/RNS from inflammation, and the resulted mutations can accumulate, which could generate cancer stem cells [3]. In OV-related cholangiocarcinoma patients, positive staining of stem cell markers, CD133 and/or Oct3/4, showed a significantly higher amount of 8-oxodG than in negative-stained cases, and these positive cases had poor prognoses. Inflammation-associated DNA damage in cancer stem-like cells may thus lead to cholangiocarcinoma development with aggressive clinical features [23].

We established a hydrogen peroxide-resistant cholangiocyte cell line, as a novel model of oxidative stress-related cholangiocarcinoma genesis [24]. Parental immortalized cholangiocyte cells (MMNK1) were treated with a relatively low concentration of hydrogen peroxide (25 μM H_2_O_2_) every day for more than 2 months, to be H_2_O_2_-resistant cells (ox-MMNK1-L, IC_50_ for H_2_O_2_ 350 μM; parental MMNK1 cells, IC_50_ for H_2_O_2_ 75 μM). The resistant cells grew more rapidly and had higher expressions of anti-oxidant enzymes such as catalase than the original parental cells. Recently, we found downregulation of early B cell factor 1 (EBF1), a tumor suppressor gene, in cholangiocarcinoma-cultured cells and ox-MMNK1-L cells compared to normal cholangiocyte cells (MMNK1) [25]. Interestingly, EBF1 knockdown in MMNK1 cells upregulated CD133 and Oct3/4 expression with a higher ability of cell migration. EBF1 downregulation was also found in OV-related cholangiocarcinoma tissues. Cholangiocarcinoma patients with low EBF1 expression and high formation of 8-oxodG had poor survival. Thus, prolonged oxidative stress due to chronic inflammation may suppress EBF1 function, leading to cholangiocarcinoma genesis via stem cell DNA damage.

Epstein-Barr (EB) virus infection is assessed as group 1 (carcinogenic to humans) by IARC. Nasopharyngeal carcinoma is a human epithelial tumor with a high prevalence in Southeast Asia and Southern China and has a strong association with EB virus infection [11, 26]. We examined stemness markers (CD44v6, ALDH1A1, CD24) in human nasopharyngeal carcinoma tissues [27]. IHC staining of CD44v6 and ALDH1A1 was observed in cancer tissues, but that of CD24 was not. Formation of the inflammation-specific DNA lesion marker 8-nitroguanine was detected in CD44v6- or ALDH1A1-positive cancer cells. Flow cytometric analysis confirmed both CD44v6- and ALDH1A1-positive cells in a small population of a human nasopharyngeal carcinoma cell line. These findings suggest the importance of 8-nitroguanine formation in nasopharyngeal cancer stem cells. In stromal areas of nasopharyngeal carcinoma tissues, α-smooth muscle actin (α-SMA)-positive cells and CD133-positive cells were detected by IHC, indicating the involvement of cancer-associated fibroblasts and hematopoietic stem/progenitor cells in neoangiogenesis [28].

### Protein damage (carbonylation)

Inflammation-derived ROS/RNS damage not only DNA but also proteins. Carbonylation is an irreversible and irreparable protein modification induced by oxidative stress. Carbonylated proteins can be detected by treatment with 2,4-dinitrophenylhydrazine (DNPH), leading to a 2,4-dinitrophenyl (DNP) hydrazone adduct [29], which is analyzed with a specific antibody for DNP hydrazine by immunoblot in 2D gel electrophoresis (2D Oxyblot) [30, 31]. We identified highly carbonylated proteins in OV-related cholangiocarcinoma tissues compared to non-cancerous tissues by 2D Oxyblot, followed by MALDI-TOF/TOF. We confirmed the highly carbonylated proteins including transferrin by using immune-precipitation and Western blotting. Interestingly, iron ion was detected in cancer tissues coincident with oxidatively damaged transferrin by Prussian blue staining and IHC. Iron release from damaged transferrin may mediate Fenton reactions and generate ROS, which may contribute to all steps of carcinogenesis [32].

### Summary of biomacromolecule damage by inflammation

**Fig. 1:**
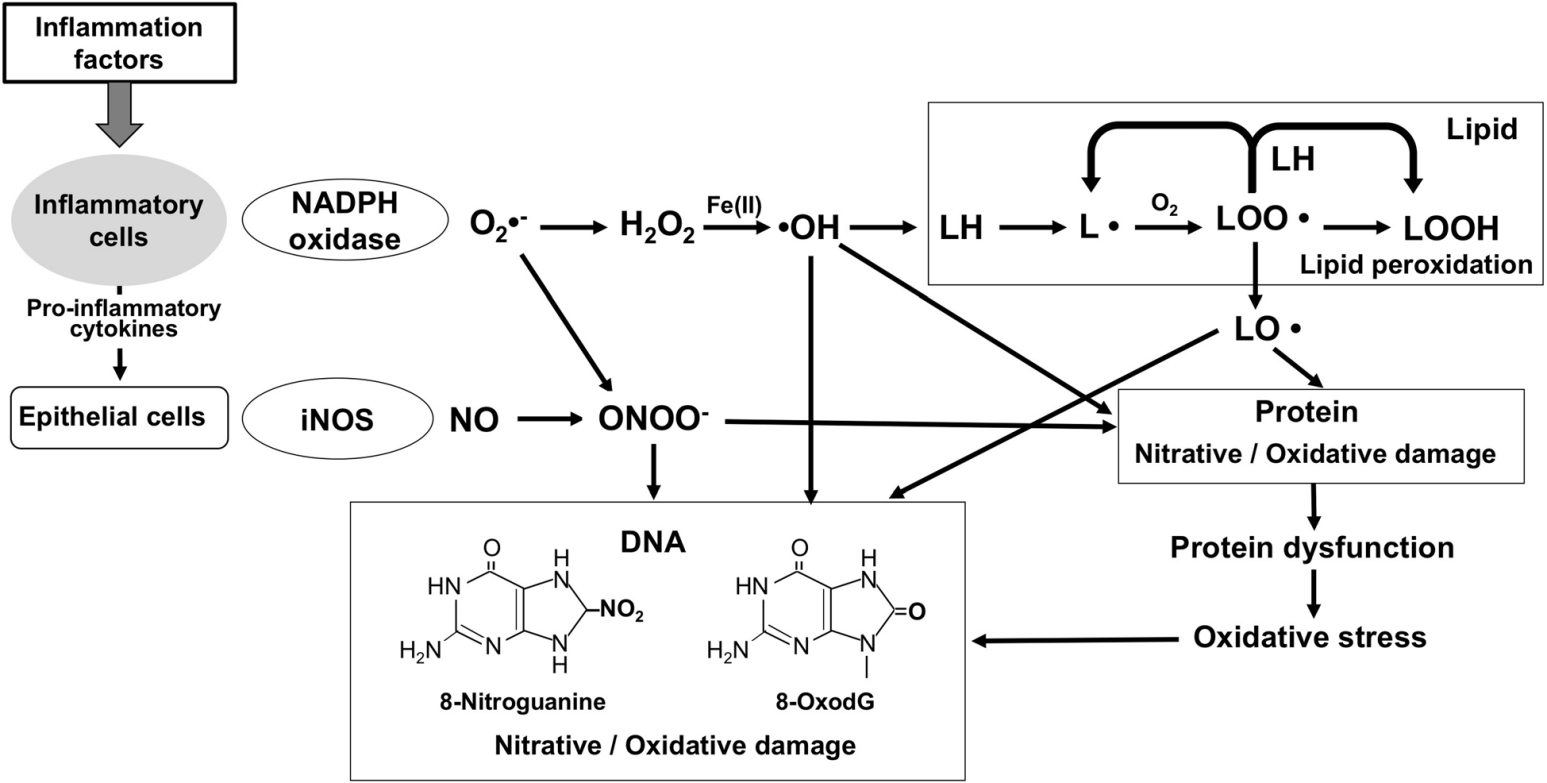
Biomacromolecule damage by inflammation

In the inflammatory microenvironment, ROS/RNS damage biomacromolecules, including DNA, proteins, and lipids, as shown in Fig. 1. Inflammation factors, such as infectious organisms and physicochemical and endogenous factors, recruit inflammatory cells to induce respiratory bursts and inflammatory cytokines. NADH oxidase and inducible nitric oxide synthase (iNOS) in inflammatory cells and epithelial cells generate superoxide (O_2_^•−^) and NO. NO reacts with O_2_^•−^ to form highly reactive peroxynitrite (ONOO^−^), causing 8-oxodG and 8-nitroguanine. H_2_O_2_ generated from dismutation of O_2_^•−^ reacts with Fe(II) to produce hydroxyl radical (•OH) in the Fenton reaction. Highly reactive •OH can attack DNA, proteins, and lipids. In addition to the above mentioned DNA and protein oxidation, inflammation also contributes to lipid peroxidation, for example, higher plasma levels of isoprostanes and malonaldehyde in cholangiocarcinoma patients [33]. Lipid peroxidation is a complex reaction; free radical attacks on polyunsaturated fatty acids yield unstable intermediates (L• and LOO•), resulting in a chain reaction that leads to a loss of membrane properties. Relatively, reactive LO• can further interact with other molecules causing protein and DNA damage. Similar to the case of iron ion from damaged transferrin, as mentioned above, oxidative stress can cause dysfunction of various molecules, forming a vicious cycle. These inflammation-related damages may play critical roles not only in cancer but also in other non-communicable diseases including cardiovascular and neurodegenerative diseases [34].

## Epigenetic alterations in inflammation-related carcinogenesis

**Fig. 2:**
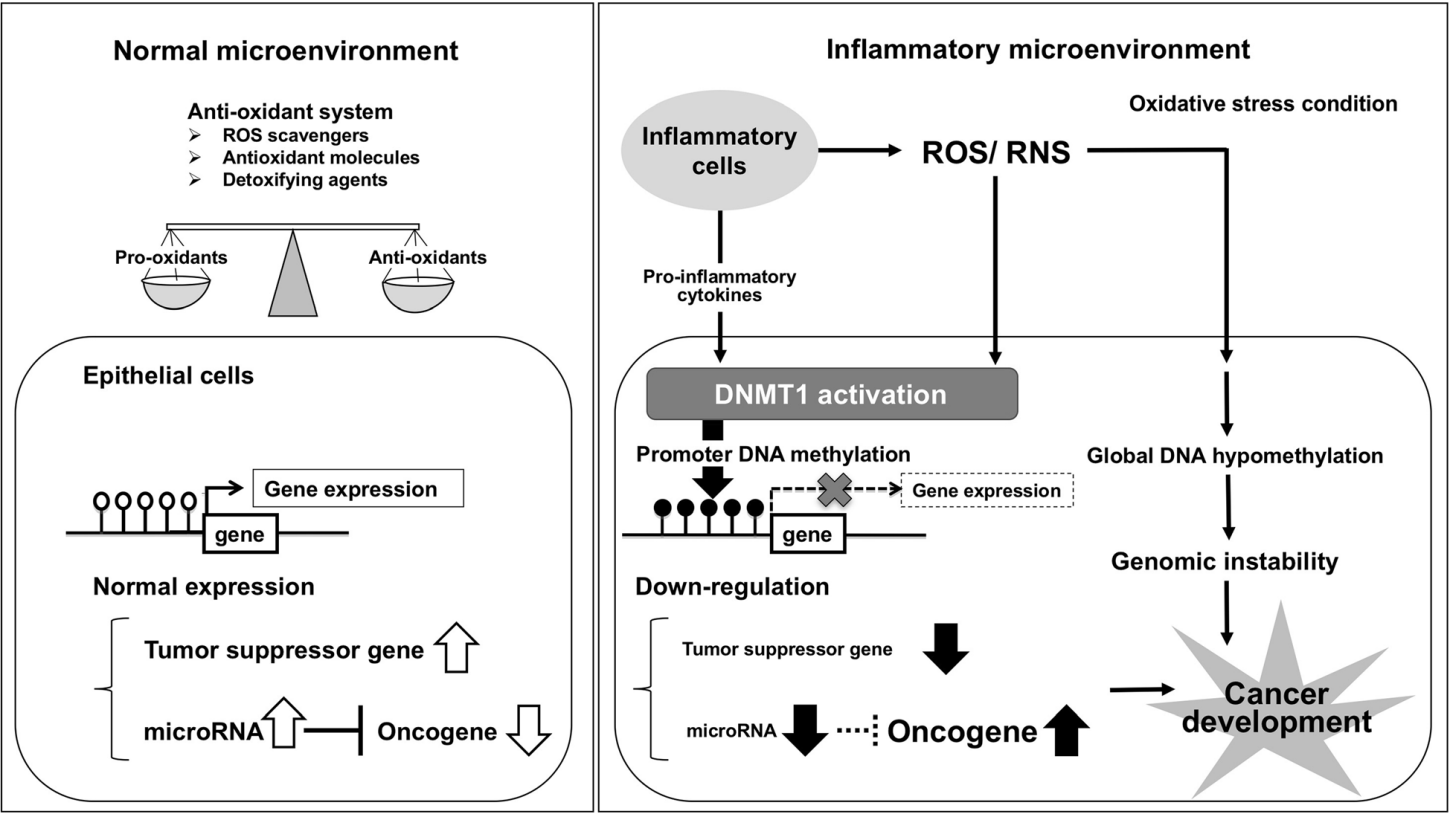
Epigenetic alterations under an inflammatory microenvironment. White circles indicate unmethylated CpG sites, and black circles show methylated CpG sites in the promoter region

Epigenetics is the phenomenon of heritable changes in gene expression that occur without a change in DNA sequence [35], through histone modification, non-coding RNA including microRNA, and DNA methylation. Under the normal microenvironment (Fig. 2, left), the anti-oxidant system works to balance pro-oxidants and anti-oxidants. Promoter cytosine-phosphate-guanine (CpG) sites of tumor suppressor genes and tumor suppressor microRNA-targeting oncogenes are hypomethylated and normally expressed. The epigenome is influenced by environmental factors such as inflammation and aging [36]. Accumulating evidence makes it increasingly clear that epigenetic silencing plays an important role in carcinogenesis via the downregulation of tumor suppressor genes and microRNAs. Under an inflammatory microenvironment (Fig. 2, right), exposure to ROS/RNS or pro-inflammatory cytokines such as interleukin 6 (IL-6) transcriptionally affects the DNA methyltransferase 1 (DNMT1) protein, resulting in enhanced DNA methylation of tumor suppressor genes and microRNAs [37]. ROS/RNS also induce global DNA hypomethylation, resulting in genomic instability. Multistage carcinogenesis consists of three steps: tumor initiation, promotion, and progression. Similar to loss-of-function by mutation, downregulation of a tumor suppressor gene by promoter DNA methylation can be the first step of carcinogenesis, “initiation.” We previously reported on ROS/RNS and IL-6 in EB virus-related human nasopharyngeal carcinoma [11]. To gain further insight regarding epigenetic changes in inflammation-related carcinogenesis, we explored the epigenetic alterations in nasopharyngeal carcinoma.

### Aberrant DNA methylation

Human malignant tumors are characterized by changes in the patterns of DNA methylation, which include a globally hypomethylated tumor cell genome and the focal hypermethylation of numerous CpG islands; many of them are associated with gene promoters [38]. Promoter hypermethylation events can lead to silencing of genes functioning in tumor-relevant pathways. Together with our collaborators at Guangxi Medical University, we examined aberrant DNA methylation of nasopharyngeal carcinoma tissues of patients in the endemic area of Southern China. We used mRNA microarrays to analyze gene expression changes in human nasopharyngeal carcinoma cell lines treated with a demethylation reagent, 5-aza-2′-deoxycytidine. Among the upregulated candidate genes, we confirmed promoter DNA hypermethylation of RRAD (RAS associated with diabetes) in biopsy specimens and cancer cell lines. Transfection of RRAD in cancer cells suppressed cell proliferation, colony formation, and migration, suggesting that RRAD has tumor-suppressive functions in nasopharyngeal carcinoma [39]. We also detected downregulation of RERG (RAS-like estrogen-regulated growth inhibitor) by DNA methylation in the cancer tissues. RERG-overexpressing cells showed significantly slower growth and less angiogenesis in tumor xenografts in nude mice [40]. DNA methylation occurs in *Helicobacter pylori* infection-related gastric cancer [41, 42] and ulcerative colitis-associated colorectal cancer [43], indicating that DNA methylation is a key event of inflammation-related carcinogenesis.

Profiling of DNA methylation across the genome is extremely important to understanding the influence of epigenetics. There has been a revolution in DNA methylation analysis technology over the past decade, and analyses that previously were restricted to specific loci can now be performed on a genome scale [44]. The main principles of DNA methylation analysis are restriction enzyme-based, affinity-based, and bisulfite deamination-based pretreatments, followed by the analytical methods such as endpoint PCR, real-time PCR, microarray, and next-generation sequencing [44]. In our early studies, we used bisulfite deamination-based methods, such as methylation-specific PCR (MSP) and bisulfite genomic sequencing (BGS) [39, 45]. Genomic DNA was treated by sodium bisulfite to deaminate unmethylated cytosine to uracil (finally detected as thymine), but methylated cytosine (5-methylcytosine) was not affected (detected as cytosine). Based on this sequence change, DNA methylation was analyzed by endpoint PCR (MSP) or Sanger sequencing of amplified bisulfited DNA (BGS). Nowadays, whole-genome bisulfite sequencing can bioinformatically lift out regions of interest, and sequencing costs are reduced. However, it remains expensive to achieve sufficient sequencing depth for high accuracy, and it is difficult to apply this technique to small samples. We recently explored methylated tumor suppressor genes using affinity-based enrichment with a methyl-CpG-binding domain protein, followed by a next-generation sequencer (methyl-capture sequencing) [46]. This method can be applied to relatively small samples at less cost. Combined with the gene expression microarray, we identified candidate genes that are silenced by promoter DNA methylation in cancer tissues. We quantified the methylation rates of the candidate genes by bisulfite amplicon sequencing (BAS, also known as amplicon bisulfite sequencing: AmpliconBS). BAS involves targeted sequencing of PCR amplicons generated from bisulfite-deaminated DNA. It is a flexible, cost-effective way to study methylation of a sample at single CpG resolution and to perform subsequent multi-target, multi-sample comparisons [47]. The BLUEPRINT consortium evaluated BAS as one of the best all-round methods for use in DNA methylation assays in large-scale validation studies, biomarker development, and clinical diagnostics [48]. We found several differentially methylated candidate genes in nasopharyngeal carcinoma tissues compared to normal nasopharynx tissues that may be useful biomarkers for cancer screening [46]. Among these candidates, we measured the methylation rates of RERG in nasopharynx biopsy specimens by using restriction enzyme-based real-time PCR, which is more convenient than BAS. The methylation rate of RERG in cancer tissues was significantly higher than that in normal tissues, with 78% sensitivity and 100% specificity to screen nasopharyngeal carcinoma [40]. It is advantageous to apply this biomarker for less-invasive specimens.

### Dysregulation of microRNA

MicroRNAs are a broad class of non-coding RNAs, 18–25 nucleotides in length in mature form, which control gene expression post-transcriptionally through binding to the 3′-untranslated region (3′-UTR) of mRNA transcripts, stimulating translational suppression or breakage of the target gene. MicroRNAs are involved in a wide range of biological processes including cell proliferation, differentiation, and apoptosis. Various microRNAs are frequently dysregulated in human cancers. We profiled microRNAs of nasopharyngeal carcinomas using microarrays and confirmed the results by quantitative RT-PCR. Among the dysregulated microRNAs in the tissues, plasma miR-497 was lower in cancer patients relative to non-cancerous control patients. Functional analyses revealed that miR-497 mimic-transfected cancer cells suppressed cell growth and migration and induced apoptosis, and showed slower tumor growth in subcutaneous xenografts. These results indicate that miR-497 has a potent tumor suppressor function and may be used as a diagnostic biomarker for nasopharyngeal carcinoma [49].

Head and neck squamous cell carcinoma (HNSCC) is a prevalent malignancy worldwide, and its risk factors such as smoking, alcohol consumption, and HPV infection, are related to inflammation. In a collaborative study with the Department of Otolaryngology and Head and Neck Surgery, Mie University, we compared microRNA expression levels between HNSCC tissues and the adjacent non-cancerous tissues by microarray. Let-7c was one of the downregulated microRNAs in HNSCC tissues. To elucidate the molecular mechanisms underlying the progression of HNSCC, we investigated the function of let-7c as a tumor suppressor. In vitro and in vivo studies revealed that let-7c negatively regulated cancer cell proliferation, migration, and epithelial-mesenchymal transition (EMT) via dysregulation of its direct target genes, insulin-like growth factor 1 receptor (IGF1R), and the high mobility group AT-hook 2 (HMGA2) [50]. Furthermore, we examined the circulating microRNA in plasma before and 6 months after treatment, revealing that plasma miR-21, miR-223, and miR-99a may serve as biomarkers to evaluate the efficacy of therapy and the prognosis of HNSCC [51]. We are currently attempting to measure circulating microRNAs at many time points after treatment to detect cancer recurrence earlier.

## Liquid biopsy

**Fig. 3:**
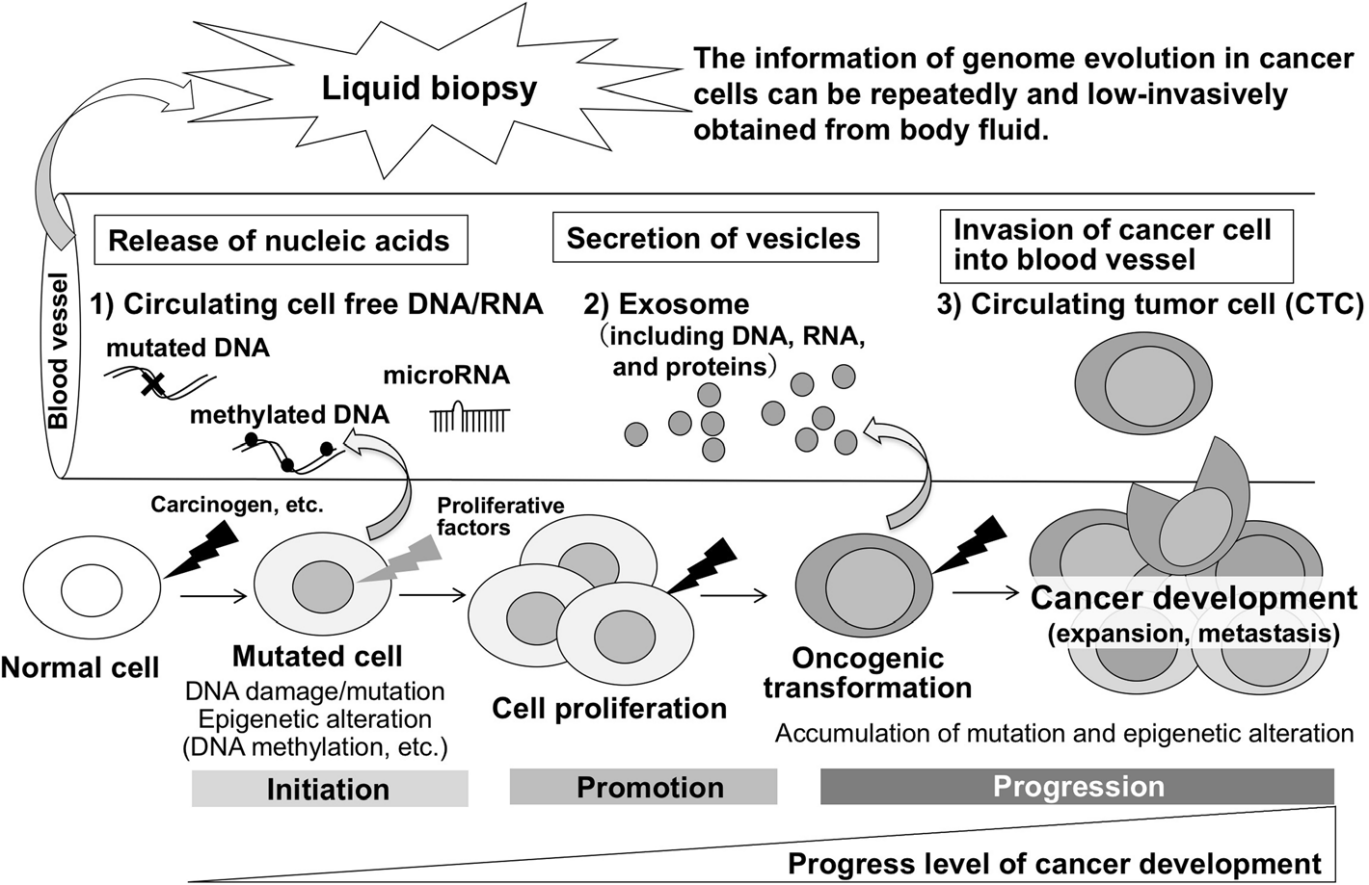
Liquid biopsy and multistage carcinogenesis

Liquid biopsy is an approach to determine the genomic profile of patients with cancer for monitoring treatment responses and to assess the emergence of therapy resistance [52]. Body fluids such as blood, urine, and cerebrospinal fluid have been shown to contain tumor-derived genetic materials and invasive tumor cells. Blood samples, as shown in Fig. 3, contain materials including circulating cell-free DNA/RNA (ccf DNA/RNA), vesicles (such as exosomes), proteins, and cells that can originate from different tissues, including cancers. The rapid turnover of cancer cells is postulated to result in the constant release of tumor-derived DNA/RNA and vesicles into the circulation, and viable tumor cells can also enter the bloodstream (circulating tumor cells, CTC). Liquid biopsy allows for repeated minimally invasive monitoring of tumor clonal evolution (mutation, epigenome alteration) during multistep carcinogenesis. In the future, liquid biopsy may be applied for early detection of cancer, if possible, at the tumor initiation step.

## Conclusion

We elucidated the molecular mechanisms of inflammation-related carcinogenesis from the aspects of DNA damage and epigenetic alteration and found several candidate biomarkers. Inflammation plays pivotal roles not only in cancer but also in neurodegenerative diseases [53], cardiovascular diseases [54], diabetes [55], and other conditions. Our previous studies indicated that oxidative stress due to inflammation causes oxidative damage to biomacromolecules and induces apoptosis in neural cells, leading to neurodegenerative diseases [31, 34, 56]. Chronic inflammation promotes genetic and epigenetic aberrations in various pathogeneses. ccf DNA/RNA, including DNA methylation and microRNA, can be biomarkers of lifestyle diseases. In the future, liquid biopsy might be a useful tool to improve preventive strategies.

## Abbreviations

3′-UTR: 3′-Untranslated region
8-OxodG: 8-Oxo-7,8-dihydro-2′-deoxyguanosine
BAS: Bisulfite amplicon sequencing
BGS: Bisulfite genomic sequencing
ccf DNA/RNA: Circulating cell-free DNA/RNA
CpG: 5′-Cytosine-phosphate-guanine-3′
CTC: Circulating tumor cells
DNMT1: DNA methyltransferase 1
DNP: 2,4-Dinitrophenyl
DNPH: 2,4-Dinitrophenylhydrazine
EB: Epstein-Barr
EBF1: Early B cell factor 1
EMT: Epithelial-mesenchymal transition
HMGA2: High-mobility group AT-hook 2
HNSCC: Head and neck squamous cell carcinoma
HPLC-ECD: An electrochemical detector coupled to HPLC
IARC: International Agency for Research on Cancer
IGF1R: Insulin-like growth factor 1 receptor
IHC: Immunohistochemistry
IL-6: Interleukin 6
iNOS: Inducible nitric oxide synthase
MSP: Methylation-specific PCR
O_2_^•−^: Superoxide
OH: Hydroxyl radical
ONOO^−^: Peroxynitrite
OV: *Opisthorchis viverrini*
RERG: RAS-like estrogen-regulated growth inhibitor
ROS/RNS: Reactive oxygen/nitrogen species
RRAD: RAS associated with diabetes
α-SMA: α-Smooth muscle actin
